# The association between C-reactive protein levels and the risk of kidney stones: a population-based study

**DOI:** 10.1186/s12882-024-03476-3

**Published:** 2024-01-27

**Authors:** Dan Liang, Chang Liu, Mei Yang

**Affiliations:** 1 Department of Endocrine, People’s Hospital of Chongqing Liang Jiang New Area, Chongqing, China; 2 School of Medicine, Nankai University, Tianjin, China

**Keywords:** C-reactive protein, Kidney stone, Obesity, Inflammation

## Abstract

**Objectives:**

The relationship between C-reactive protein (CRP) and the risk of developing kidney stones is unclear, and we aimed to assess the association between CRP and kidney stones in US adults.

**Methods:**

We used data from NHANES 2007–2010, and we excluded participants who were under 18 years of age and lacked data on CRP and kidney stones. Finally, we included a total of 11,033 participants and performed weighted multivariate regression analysis and subgroup analysis to assess the independent relationship between CRP and kidney stones.

**Results:**

The mean prevalence of kidney stones among the participants was 9.8%. Notably, as CRP levels increased, the prevalence of kidney stones exhibited a corresponding rise across quartiles (Kidney stones: Quartile 1: 7.59%; Quartile 2: 8.77%; Quartile 3: 9.64%; Quartile 4: 10.89%). CRP was positively associated with the risk of kidney stones (Model 1: OR = 1.09, 95% CI: 1.01–1.18, *p* = 0.03; Model 2: OR = 1.09, 95% CI: 1.00–1.18, *p* = 0.03, Model 3: OR = 1.14, 95%CI: 1.02–1.26, *p* = 0.04). Participants in the highest CRP quartile experienced a 69% increased risk of kidney stones compared to those in the lowest quartile (OR = 1.64, 95% CI: 1.04–2.59, *p* = 0.03). Notably, interaction tests revealed that gender, BMI, diabetes, hypertension, CKD and smoking or alcohol consumption status did not significantly influence the association between CRP and kidney stones.

**Conclusions:**

Our findings reveal a significant association between higher CRP levels and an increased risk of kidney stones. In clinical practice, heightened awareness of CRP as a potential biomarker could aid in risk assessment and management strategies for kidney stone patients.

**Supplementary Information:**

The online version contains supplementary material available at 10.1186/s12882-024-03476-3.

## Introduction

Kidney stones, mineral deposits in the renal pelvis and calyces, pose a growing public health challenge globally. The rise in prevalence may be attributed to improved detection of asymptomatic stones [1]. In the United States, adult men exhibit a prevalence of 10.9%, slightly lower in adult women at 9.5%. The prevalence has increased from 6.5% in 2007–2008 to 9.4% in 2017–2018 [2]. Notably, kidney stone recurrence is substantial, with rates reaching 35% within 5 years and 52% within 10 years [3]. Calcium oxalate stones are the most common type, followed by hydroxy phosphate and calcium urate stones [4]. Additionally, the occurrence of kidney stones may be linked to an elevated risk of adverse renal outcomes, including end-stage renal disease, chronic kidney disease, and even a single episode of kidney stones during follow-up [5].

C-reactive protein (CRP), an acute-phase reactant in the circulation, is induced in the liver by pro-inflammatory factors. It plays a role in mediating tissue damage, inflammatory responses, and infections [6, 7]. CRP is a nonspecific inflammatory marker that rapidly elevates in inflammatory responses and has been linked to an increased risk of cardiovascular disease and cancer [8, 9].

CRP exhibits a specific relationship with the pathological state of kidney disease, serving as a biomarker for renal pathology [10, 11]. In preterm neonates with acute kidney injury (AKI), elevated CRP emerged as a critical factor influencing AKI [12]. The association of CRP with pyelonephritis caused by obstructive renal stones has been demonstrated [13]. CRP is also considered to be a valid parameter for assessing whether to undergo urethral stenting in patients with renal colic caused by urinary stones, and is more informative than serum creatinine and leukocyte levels [14]. Studies have weakly correlated CRP with renal stone formation in Japanese men [15]. The mechanism of the inflammatory response in kidney stone formation is not fully understood but may involve the production of reactive oxygen species and the activation of inflammasomes [16].

Furthermore, the inflammatory response observed in kidney stones may be associated with a microinflammatory state within the kidney. Microinflammation is characterized as a systemic, chronic, non-dominant mild inflammatory change induced by non-pathogenic microbial infections [17]. This condition is primarily marked by elevated pro-inflammatory factors in the circulatory system, including CRP and interleukin (IL)-6, along with activation of the mononuclear phagocyte system. However, the precise mechanism underlying microinflammation remains unclear and may involve abnormal macrophage phenotype, increased macrophage infiltration in adipose tissue, and neutrophil apoptosis leading to myeloperoxidase release [17–19]. Hence, our investigation aimed to determine whether there exists a correlation between CRP levels and the risk of developing kidney stones.

## Methods

### Study population

We obtained data from NHANES, a cross-sectional study aimed to evaluate the health and nutrition status of the US population administered by the National Center for Health Statistics (NCHS) of US Center for Disease Control and Prevention (CDC). All NHANES data are publicly available at https://www.cdc.gov/nchs/nhanes/. The NHANES survey is a national research program conducted in a 2-year repeated cycle with continuously updated survey data. The NHANES study design uses a complex stratified, multistage probability sampling method to assess the health and nutrition status of the U.S. population, and it recruits participants with a degree of representativeness. The NHANES program was approved by the NCHS Ethics Review Board, and all participants have signed informed consent.

**Fig. 1:**
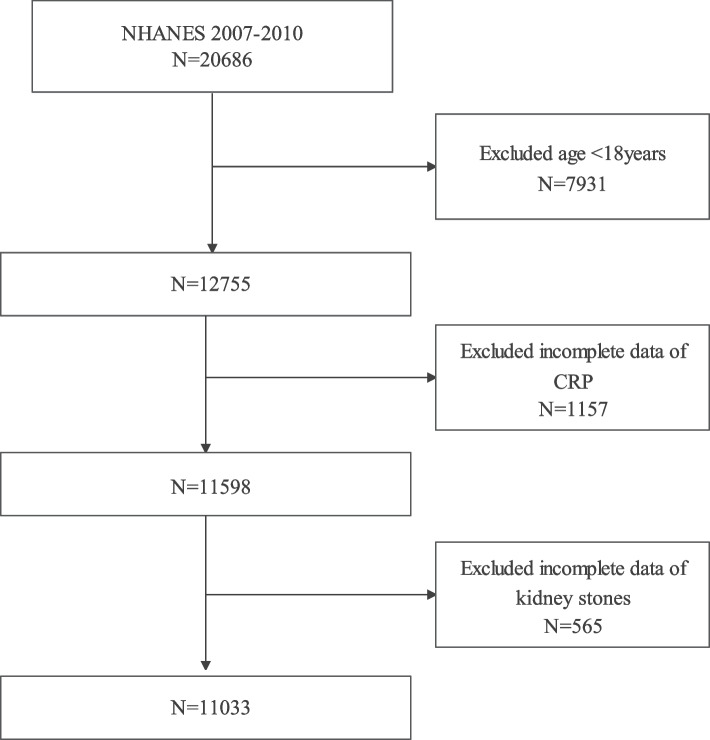
Flowchart of the sample selection from National Health and Nutrition Examination Survey (NHANES)

We used data from 2007–2010 NHANES database, becaues only these two cycles include data on both kidney stones and CRP, and initially we included a total of 20,686 participants, excluding those aged under 18 years (*N* = 7931) and lacking data on CRP (*N* = 1157) and kidney stones (*N* = 565), resulting in the inclusions of 11,033 participants in our study (Fig. 1).

### Exposure and outcome definitions

CRP was employed as an exposure variable. Blood specimens underwent processing, storage, and shipment to the University of Washington in Seattle, WA. Quantification of CRP was accomplished using latex-enhanced nephelometry. Particle-enhanced assays relied on the interaction between a soluble analyte and the corresponding antigen or antibody bound to polystyrene particles. For the quantification of CRP, particles comprising a polystyrene core and a hydrophilic shell were utilized to covalently attach anti-CRP antibodies. A diluted test sample was combined with latex particles coated with mouse monoclonal anti-CRP antibodies. CRP present in the test sample formed an antigen–antibody complex with the latex particles. Automatic blank subtraction was applied, and CRP concentrations were determined using a calibration curve. These assays were conducted using a Behring Nephelometer for quantitative CRP determination.

The primary outcome of the analysis was the response to the question, "Have you ever experienced kidney stones?" If a participant answered affirmatively, they were classified as having nephrolithiasis. The occurrence of kidney stones was designated as the outcome variable."

### Covariates

Covariates in our study included age (years), gender (female/male), race (Mexican American/Non-Hispanic Black/Non-Hispanic White/Other races), educational level (< 9th grade/9-11th grade/college graduate or above/high school graduate/some college or AA degree), poverty-to-income ratio (PIR), body mass index (BMI), total cholesterol, triglyceride, low density lipoprotein (LDL), high density lipoprotein (HDL), estimated glomerular filtration rate (eGFR), urine albumin creatinine ratio (uACR), serum calcium, serum uric acid, total water drink intake, total energy intake, drinking status, smoke status, diabetes (DM), hypertension, chronic kidney disease (CKD), and total intakes of calcium, phosphate, sodium, potassium, protein, fat, and carbohydrate. Hypertension was defined based on a self-reported diagnosis of hypertension, diastolic blood pressure ≥ 90 mmHg or systolic blood pressure ≥ 140 mmHg, or the use of antihypertensive medications [20]. Diabetes mellitus was defined base on a self-reported diagnosis of diabete mellitus, 2-h plasma glucose ≥ 200 mg/dL in an oral glucose tolerance test, HbAlc ≥ 6.5%, use of oral hypoglycemic agents, or fasting glucose ≥ 126 mg/dL [21]. BMI was classified as < 25, 25–29.9 and ≥ 30 kg/m2, which corresponded to normal weight, overweight and obese populations for all participants. Dietary intake of calcium, phosphate, sodium, potassium, protein, fat, and carbohydrate was calculated using the University of Texas Food Intake Analysis System and the US Department of Agriculture Survey Nutrient Database. The assessment of dietary nutrients intake was derived from the total nutrient intake file, which contains comprehensive nutrient data encompassing all foods and beverages consumed by participants. Nutrients obtained from dietary supplements or medications were excluded from the nutrient estimates. Chronic kidney disease (CKD) is characterized by albuminuria or low eGFR, as defined in Kidney Disease: Improving Global Outcomes 2012 [22]. All detailed measurement processes of study variables were publicly available at www.cdc.gov/nchs/nhanes/.

### Statistical analysis

All statistical analyses were conducted according to Centers for Disease Control and Prevention (CDC) guidelines, and an appropriate NHANES sampling weight was applied and accounted for the complex multistage cluster survey design in the analysis. Continuous variables were expressed as means and standard errors, and categorical variables were expressed as percentages. Differences between groups by CRP (quartiles) were assessed using a weighted Student's t-test (continuous variables) or a weighted chi-square test (categorical variables). Multivariate logistic regression models were used to explore the independent relationship between CRP and renal calculi in three different models. In Model 1, no covariateds were adjusted. Model 2 was adjusted for gender, age, race and educational levels. Model 3 was adjusted for gender, age, race, educational levels, PIR, BMI, triglyceride, total cholesterol, LDL, HDL, serum uric acid, serum calcium, eGFR, uACR, total water drink intake, total energy intake, total intakes of calcium, phosphorus, sodium, potassium, protein, fat, and carbohydrate, diabetes, hypertension, CKD, drinking and smoking status. Subgroup analysis stratified by gender, diabetes, hypertension, BMI, drinking and smoking status were also performer by stratified multivariate regression analysis. In addition, an interaction term was added to test the heterogeneity of associations between the subgroups using log likelihood ratio test model. A two side *p* < 0.05 was considered statistically significant. All analyses were preformed using R version 4.2.1 (http://www.R-project.org, The R Foundation).

## Results

### Baseline characteristics of participants

The demographic baseline characteristics of included participants were shown in Table 1. A total of 11,033 participants were enrolled in this study, of whom 51.81% were female and 48.19% were male. The average age of the participants was 47.01 ± 0.32 years. The mean value of CRP was 0.39 ± 0.01 mg/dl. The CRP ranges for quartiles 1–4 were 0.01–0.08, 0.08–0.19, 0.19–0.46, 0.46–20 mg/dl, respectively. The normal range for C-reactive protein (CRP) is defined as 0–1 mg/dl. Notably, only participants in the highest quartile exhibited CRP levels above this normal range. The average prevalence of kidney stones was 9.8%. Notably, participants in Quartile 4 of CRP exhibited a higher prevalence of kidney stones (10.89%, *p* = 0.01) compared to Quartiles 1–3. Participants in Quartile 4 of CRP were more likely to be female, Non-Hispanic White and obese, demonstrated elevated levels of serum uric acid, uACR, triglyceride and LDL, coupled with lower levels of eGFR, and had a higher likelihood of smoking, diabetes and hypertension. Moreover, participants in Quartile 4 of CRP reported lower intakes of calcium, phosphate, sodium, potassium, protein, fat and carbohydrate. In addition, individuals in the highest CRP quartile also exhibited lower energy intake.

**Table 1:** Baseline characteristics of participants

CRP (mg/dl)	All participants	Q1 (0.01–0.08)	Q2 (0.08–0.19)	Q3 (0.19–0.46)	Q4 (0.46–20)	*P* value
Demographic variables						
Age (year)	47.01 (0.32)	44.22 (0.40)	48.03 (0.51)	48.43 (0.40)	48.29 (0.55)	** < 0.0001**
Gender (%)						** < 0.0001**
Female	51.81 (0.02)	46.70 (0.90)	45.68 (1.15)	53.61 (1.35)	63.66 (1.01)	
Male	48.19 (0.02)	53.30 (0.90)	54.32 (1.15)	46.39 (1.35)	36.34 (1.01)	
Races (%)						** < 0.0001**
Mexican American	8.50 (0.01)	6.64 (0.93)	8.16 (1.32)	10.02 (1.64)	9.82 (1.92)	
Non-Hispanic Black	10.57 (0.01)	8.44 (0.80)	9.33 (1.03)	10.86 (1.10)	14.62 (1.64)	
Non-Hispanic White	69.39 (0.05)	71.30 (2.12)	70.43 (2.55)	68.80 (2.84)	66.23 (3.23)	
Other races	11.54 (0.01)	13.62 (1.48)	12.08 (1.67)	10.33 (1.21)	9.34 (1.27)	
Education levels (%)						** < 0.0001**
< 9th Grade	6.69 (0.01)	5.45 (0.43)	6.77 (0.66)	7.27 (0.75)	7.73 (0.81)	
9-11th Grade	12.96 (0.01)	10.32 (0.75)	12.51 (1.01)	14.54 (0.81)	15.44 (1.12)	
High School Graduate	23.93 (0.02)	20.51 (0.89)	24.30 (1.61)	25.62 (1.39)	26.52 (1.07)	
Some College or AA degree	29.63 (0.01)	29.14 (1.12)	29.06 (1.33)	29.10 (1.27)	31.52 (1.26)	
College Graduate or above	26.79 (0.01)	34.58 (1.89)	27.36 (1.34)	23.46 (1.71)	18.78 (1.48)	
PIR (%)						** < 0.0001**
< 1	13.18 (0.01)	11.88 (0.73)	13.31 (1.12)	14.45 (1.01)	18.30 (1.36)	
1–4	45.30 (0.02)	46.90 (1.73)	48.15 (1.70)	49.89 (1.82)	51.56 (1.46)	
> 4	34.10 (0.02)	41.22 (1.58)	38.54 (1.46)	35.66 (1.92)	30.15 (1.92)	
BMI (%)						** < 0.0001**
Normal weight	31.10 (0.01)	53.33 (1.10)	30.26 (1.37)	20.92 (1.00)	13.57 (0.74)	
Overweight	33.10 (0.01)	34.56 (0.98)	39.66 (1.10)	35.00 (0.99)	24.08 (1.14)	
Obesity	34.30 (0.02)	12.10 (0.75)	30.08 (1.04)	44.08 (1.00)	62.34 (1.21)	
Smoke (%)						** < 0.0001**
Former	24.42 (0.01)	23.27 (0.89)	24.05 (1.21)	24.99 (1.01)	25.86 (1.18)	
Never	54.11 (0.02)	58.24 (1.32)	55.26 (1.70)	52.02 (1.21)	49.40 (1.78)	
Now	21.44 (0.01)	18.49 (0.98)	20.69 (1.19)	22.99 (1.15)	24.74 (1.31)	
Alcohol user (%)						** < 0.0001**
Yes	66.25 (0.03)	70.48 (1.11)	68.79 (1.37)	63.67 (2.01)	60.37 (1.76)	
No	33.75 (0.02)	29.52 (1.11)	31.21 (1.37)	36.33 (2.01)	39.63 (1.76)	
DM (%)	13.59 (0.01)	8.27 (0.78)	11.66 (0.64)	15.28 (0.79)	22.17 (1.18)	** < 0.0001**
Hypertension (%)	35.70 (0.02)	25.22 (1.20)	36.12 (1.27)	39.21 (1.71)	46.27 (1.33)	** < 0.0001**
CKD (%)	13.47 (0.01)	9.66 (0.51)	12.65 (0.87)	14.50 (0.77)	19.61 (0.91)	** < 0.0001**
Kidney stone (%)	9.08 (0.01)	7.59 (0.47)	8.77 (0.54)	9.64 (0.95)	10.89 (0.61)	**0.01**
Laboratory examination variables						
Serum uric acid (umol/L)	325.32 (1.42)	306.72 (1.95)	327.83 (2.17)	332.89 (2.18)	340.74 (2.78)	** < 0.0001**
Serum creatinine (mg/dl)	0.88 (0.01)	0.87 (0.01)	0.89 (0.01)	0.87 (0.01)	0.88 (0.01)	0.14
Total cholesterol (mmol/L)	5.09 (0.02)	4.92 (0.02)	5.15 (0.03)	5.21 (0.04)	5.13 (0.03)	** < 0.0001**
Triglyceride (mmol/L)	1.49 (0.02)	1.25 (0.03)	1.48 (0.04)	1.66 (0.04)	1.67 (0.04)	** < 0.0001**
LDL (mmol/L)	3.00 (0.02)	2.83 (0.03)	3.05 (0.03)	3.06(0.33)	3.11 (0.03)	** < 0.0001**
HDL (mmol/L)	1.36 (0.01)	1.48 (0.01)	1.36 (0.02)	1.31 (0.01)	1.25 (0.01)	** < 0.0001**
uACR (mg/g)	31.65 (2.90)	24.77 (5.42)	23.08 (3.05)	26.14 (3.09)	56.97 (9.59)	**0.02**
eGFR (mL/min/1.73m2)	94.99 (0.55)	97.13 (0.56)	93.87 (0.65)	93.98 (0.77)	94.29 (0.79)	** < 0.0001**
CRP (mg/dl)	0.39 (0.01)	0.04 (0.01)	0.14 (0.01)	0.30 (0.01)	1.24 (0.02)	** < 0.0001**
Nutrient intake variables						
Calcium (mg/d)	982.52 (11.70)	1024.60 (19.78)	1011.13 (19.74)	962.51 (18.73)	914.61 (14.20)	** < 0.0001**
Phosphorus (mg/d)	1390.92 (11.87)	2856.69 (42.44)	2826.23 (37.63)	2688.34 (41.93)	2490.04 (39.61)	** < 0.0001**
Sodium (mg/d)	3568.56 (29.82)	3719.60 (48.67)	3647.11 (50.41)	3509.56 (44.89)	3336.42 (48.81)	** < 0.0001**
Potassium (mg/d)	2729.11 (25.09)	2856.69 (42.44)	2826.23 (37.63)	2688.34 (41.93)	2490.04 (39.61)	** < 0.0001**
Total water drink (g/d)	409.12 (23.64)	418.52 (29.61)	400.32 (32.41)	390.12 (26.84)	426.30 (25.33)	0.5
Protein (g/d)	83.27 (0.77)	86.05 (1.13)	86.43 (1.18)	81.90 (1.26)	77.47 (1.00)	** < 0.0001**
Fat (g/d)	81.93 (0.88)	85.78 (1.54)	83.89 (1.33)	80.52 (1.15)	76.59 (1.10)	** < 0.0001**
Carbohydrate (g/d)	261.46 (2.02)	273.34 (3.59)	265.25 (3.88)	257.10 (2.95)	245.46 (3.13)	** < 0.0001**
Total energy intake (g/d)	9.08 (0.01)	2262.19 (27.10)	2230.54 (30.11)	2126.39 (24.62)	2015.01 (22.45)	** < 0.0001**

*BMI* Body mass index, *PIR* Ratio of family income to poverty, *uACR* Urine albumin creatinine ratio, *LDL* Low density lipoprotein, *HDL* High density lipoprotein, *DM* Diabetes, *CKD* Chronic kidney disease, *CRP* C-reactive protein, *eGFR* estimated Glomerular Filtration Rate

### Association between CRP and the risk of kidney stones

The association between CRP and the risk of kidney stones was examined, as presented in Table 2. A positive association was observed between CRP and the risk of kidney stones (OR = 1.09, 95% CI: 1.01–1.18, *p* = 0.03) in the unadjusted model. In Model 2, we adjusted for age, gender and race, and the positive association between CRP and the risk of kidney stones persisted (OR = 1.09, 95% CI: 1.00–1.18, *p* = 0.03).The positive association between CRP and the risk of kidney stones still remained stable in the fully adjusted model (OR = 1.14, 95%CI: 1.02–1.26, *p* = 0.04).

**Table 2:** Association of C-reactive protein levels with kidney stones

CRP levels	OR^1^(95% CI^2^), *p*-value		
	Model 1	Model 2	Model 3
Kidney stone			
Continous	1.09 (1.01, 1.18), ***p* = 0.03**	1.09 (1.00, 1.18), ***p* = 0.03**	1.14 (1,02, 1.26), ***p* = 0.04**
Categories			
Quartile 1	Reference	Reference	Reference
Quartile 2	1.17 (0.95, 1.44), *p* = 0.13	1.07 (0.87, 1.33), *p* = 0.49	1.10 (0.74, 1.64) *p* = 0.62
Quartile 3	1.30 (1.00, 1.69), ***p* = 0.04**	1.34 (1.04, 1.64), ***p* = 0.02**	1.54 (1.07, 2.01), *p* = 0.32
Quartile 4	1.49 (1.20, 1.84), ***p* < 0.001**	1.53 (1.22, 1.92), ***p* < 0.001**	1.64 (1.04, 2.59), ***p* = 0.03**

*OR* Odd ratio, *95%CI*: 95% confidence interval

Model 1, no covariates were adjusted

Model 2 was adjusted for gender, age, race

Model 3 was adjusted for gender, age, race, educational levels, poverty-to-income ratio, BMI, triglyceride, total cholesterol, HDL, LDL, serum uric acid, serum calcium, eGFR, uACR, total water drink, total intakes of calcium, phosphorus, sodium, potassium, total intakes of protein, carbohydrate, fat, total energy intake, diabetes, hypertension, CKD, drinking and smoking status

To conduct a sensitivity analysis, we transformed CRP from a continuous variable to a categorical variable (quartiles), participants in the highest CRP quartile demonstrated a substantial 64% increase in the risk of kidney stones compared to those in the highest CRP quartiles (OR = 1.64, 95%CI: 1.04–2.59, *p* = 0.03).

### Subgroup analysis

To delve deeper into the relationship between CRP and the risk of kidney stones, subgroup analyses were conducted based on gender, BMI, smoking or alcohol consumption, and the presence of hypertension, diabetes and CKD (Table 3). In subgroup analysis, we observed a positive association between CRP and increased risk of kidney stones in the obese subgroup (*p* = 0.031), but this positive association did not meet statistical significance in overweight participants (*p* = 0.284), however, no correlation with the p for interaction that met statistical significance was observed, indicating that this correlation was not dependent on gender, BMI, smoking, alcohol consumption, hypertensio, diabetes and CKD, as all *p* for interaction > 0.05. This result highlighted the robustness of the correlation across various subgroups.

**Table 3:** Subgroup analysis

Kidney stone	OR (95%CI)	*P* for trend	*P* for interaction
Gender			0.880
Female	1.11 (0.80, 1.54)	0.532	
Male	1.14 (0.89, 1.44)	0.287	
Hypertension			0.116
Yes	1.07 (0.85, 1.36)	0.558	
No	1.21 (0.95, 1.55)	0.120	
Diabetes			0.198
Yes	1.16 (0.75, 1.44)	0.718	
No	0.94 (0.65, 1.35)	0.153	
Smoke			0.690
Never	1.15 (0.94, 1.41)	0.179	
Former	1.24 (0.79, 1.95)	0.346	
Now	1.11 (0.85, 1.46)	0.427	
Alcohol user			0.516
Yes	1.17 (0.89, 1.54)	0.251	
No	1.15 (0.87, 1.53)	0.325	
BMI			0.054
Normal weight	0.85 (0.55, 1.33)	0.471	
Overweight	1.11 (0.91, 1.35)	0.284	
Obesity	1.34 (1.04,1.74)	**0.031**	
CKD			0.440
Yes	0.95 (0.82, 1.11)	0.522	
No	1.22 (0.93, 1.60)	0.151	

Subgroup analysis. OR for the association between CRP levels and kidney stone. All presented covariates were adjusted (as Model 3) except the corresponding stratification variable

## Discussion

In this large cross-sectional study including 11,033 US adults, we found a positive association between CRP levels and the risk of kidney stones in unadjusted models and in models adjusted for age, gender, race and educational levels. Higher CRP levels were associated with a higher incidence of kidney stones. In addition, we found that higher CRP quartiles showed a higher risk of kideney stone development compared to the lowest CRP quartiles (OR of 1.10, 1.54 and 1.64 for Quartiles 2, 3, and 4, respenctively). This suggested that the levels of CRP increased the risk of developing kidney stones. We also observed that this association was similar in subgroups stratified by gender, race, BMI, hypertension, diabetes, smoking and drinking status, which may suggest that this association can be applied across population settings. In conclusion, our finding from this study suggested that clinicians should focus on CRP levels in patients with kidney stones.

Kidney stones, a prevalent global condition affecting approximately 14.8% of the population [23], are associated with various risk factors including obesity, hypertension, and metabolic syndrome [24]. Emerging evidence suggests a connection between kidney stone formation and autoimmune diseases [25]. CRP, a pentameric biomarker indicating acute phase inflammation [6], can also served as a valuable biomarker of chronic low-grade inflammation [26, 27]. Elevated CRP levels have been linked to chronic systemic diseases, such as type 2 diabetes, contributing to endothelial dysfunction and vascular remodeling in diabetes [28]. Moreover, increased CRP is independently associated with higher vascular and all-cause mortality in individuals with type 2 diabetes [29]. While some studies questioned the causal link between elevated CRP and cancer risk, CRP levels remained a predictive marker for lung cancer in current smokers [30].

The relationship between CRP and the urinary system is multifaceted. CRP plays a crucial role in predicting the failure of spontaneous stone expulsion in lower urinary tract stones and serves as a marker for urinary tract obstruction in renal colic patients [14, 31]. A notable fivefold increase in CRP serves as a significant predictor of acute renal colic combined with urinary tract infection [32]. Furthermore, CRP emerges as a prognostic indicator for overall mortality in patients with muscle-invasive bladder cancer undergoing radical cystectomy [33]. In the context of diabetic nephropathy, CRP exacerbates its development by inhibiting autophagy in podocytes through the suppression of signaling in the C3a/C3aR axis [34]. Additionally, CRP levels significantly rise with increasing lower urinary tract dysfunction [35].

The precise mechanism underlying the positive association between CRP and kidney stones remains unclear. The formation of kidney stones is intricately linked to several processes, including the production of reactive oxygen species, inflammasome activation, and increased expression of molecules associated with the inflammatory cascade, such as bone bridging proteins and matrix Gla proteins [16]. Macrophages play a pivotal role in kidney stone formation, with classically activated pro-inflammatory macrophages being implicated, while the downregulation of anti-inflammatory macrophages inhibits kidney stone formation [36]. Calcium oxalate crystals, a key component in kidney stones, induce an inflammatory response by activating the NLR family pyrin domain containing 3 inflammasome (NLRP3), resulting in the release of pro-inflammatory cytokines IL-1β and IL-18 [37]. Moreover, calcium oxalate monohydrate compounds trigger pro-inflammatory signaling and oxidative stress in renal tubular epithelial cells, crucial steps in kidney stone formation [38]. Mitochondrial dysfunction and redox imbalance in patients with calcium oxalate kidney stones can lead to impaired clarity of stone crystals [39]. Elevated oxalate levels mediated by hydroxyproline can result in increased inflammatory markers in rats and reprogram signaling pathways in macrophages [40]. The activation of the NF-kB/p38 signaling pathway is implicated in crystal aggregation within renal tissues, enhancing the expression of oxidative damage and inflammation-associated proteins [41]. These collective findings underscore the central role of inflammation and oxidative stress in the formation of kidney stones. Inflammation is not merely a consequence but a key pathogenic factor in kidney stones. Despite its significant importance in kidney stone patients, the immune and inflammatory responses to kidney stone disease remain an area of ongoing exploration.

In our current investigation, CRP emerges as a promising biomarker for identifying the risk of kidney stones. Previous studies have also explored additional biomarkers capable of reflecting the risk of kidney stones. Notable among these are urinary biomarkers such as neutrophil gelatinase-associated lipocalin (NGAL) and kidney injury molecule-1 (KIM-1), which have demonstrated diagnostic potential in patients with kidney stones [42]. However, their utility may be compromised by potential confusion with other causes of kidney injury, such as obstructive nephropathy. Further investigation reveals that urinary hippuric acid (HA) and citrate can serve as informative biomarkers associated with fruit and vegetable intake in individuals with nephrolithiasis [43]. Additionally, the higher Neutrophil–lymphocyte ratio (NLR) is found to be closely linked to kidney stones and the number of stones passed [44]. These findings underscore the importance of elucidating the significant role that potential biomarkers can play in the screening and prevention of kidney stones in future clinical practice.

While our study drew upon data from NHANES, a national population-based sample survey, and we implemented adjustments for confounding covariates to minimize bias, it is essential to acknowledge the study's limitations. Primarily, the cross-sectional design of our study precludes establishing a causal relationship between C-reactive protein (CRP) and the risk of kidney stones. To address this limitation and provide more robust insights, a longitudinal study with a larger sample size would be invaluable. Despite our efforts to adjust for covariates, it is important to note that complete exclusion of the effects of all potential confounders cannot be guaranteed. The intricacies of individual health profiles and external factors may introduce variability that could influence the observed associations. Moreover, the design of the NHANES study limited the collection of certain critical variables, including urinary parameters such as urinary flow rate, urinary acidity/base, and the presence of urinary crystals. Similarly, clinical variables like the age of onset and family history were not included in the NHANES database, and unfortunately, we were unable to acquire this additional information. The absence of these variables limits the comprehensiveness of our analysis and prevents a more thorough exploration of potential associations. Furthermore, the lack of data on participants' urinary tract infections poses a constraint on our ability to investigate the relationship between C-reactive protein (CRP) and kidney stones specifically in individuals without urinary tract infections. This information could have provided valuable insights into the interplay between CRP and kidney stones in a more defined subgroup. Despite these limitations, our study contributes to the existing knowledge base, and future research with more extensive data collection could further elucidate these relationships. Finally, the reliance on self-reported data for kidney stones introduces potential intraperson variability and recall bias. This limitation underscores the need for caution in the interpretation of our findings. Additionally, the absence of data on the components of kidney stones precluded a more in-depth analysis of the relationship between CRP and specific types of kidney stones. Future research endeavors with more comprehensive datasets could provide a more nuanced understanding of these relationships.

## Conclusions

Our study demonstrated that increased CRP levels were associated with an elevated likelihood of kidney stones. In addition, our study also suggested that the positive correlation between CRP and kidney stones is similar across gender, BMI, hypertension, CKD and DM status, and the presence or absence of smoking or alcohol consumption, and may be applicable to different population settings. However, further large prospective studies are still needed to validate our findings.

### Supplementary Information

**Additional file 1.**

## Data Availability

Data described in the manuscript, codebook, and analytic code will be made publicly and freely available without restriction at www.cdc.gov/nchs/nhanes/.
