# Supplementary material for: The association between C-reactive protein levels and the risk of kidney stones: a population-based study
Source: BMC Nephrol. 2024 Jan 27;25:39. doi: 10.1186/s12882-024-03476-3 (PMC10822160; doi:10.1186/s12882-024-03476-3)
Supplement: Supplementary file 1 — Additional file 1. [file 12882_2024_3476_MOESM1_ESM.pdf]

National Center for Health Statistics

National Health and Nutrition Examination Survey

NCHS Ethics Review Board (ERB) Approval*

Survey Name/Date	NCHS IRB/ERB Protocol Number or Description
NHANES 2021-2022	Protocol #2021-05
NHANES 2019-2020	Protocol #2018-01
NHANES 2017-2018	Protocol #2018-01 (Effective beginning October 26, 2017)
	Continuation of Protocol #2011-17 (Effective through October 26, 2017)
NHANES 2015-2016	Continuation of Protocol #2011-17
NHANES 2013-2014	Continuation of Protocol #2011-17
NHANES 2011-2012	Protocol #2011-17
NHANES 2009-2010	Continuation of Protocol #2005-06
NHANES 2007-2008	Continuation of Protocol #2005-06
NHANES 2005-2006	Protocol #2005-06
NHANES 1999-2004	Protocol #98-12
NHANES III	Institutional Review Board (IRB) approval and documented consent was obtained from participants
NHANES II	Underwent internal human subjects review, but IRB approval using current standards was not obtained.
NHANES I	Underwent internal human subjects review, but IRB approval using current standards was not obtained.
NHES	Underwent internal human subjects review, but IRB approval using current standards was not obtained.

* In 2003, the NHANES Institutional Review Board (IRB) changed its name to the NCHS Research Ethics Review Board (ERB). In 2018, the name was changed from NCHS Research Ethics Review Board to NCHS Ethics Review Board.

Page last reviewed: August 24, 2022
